# Effect of individualized psychological interventions on the psychological state and pregnancy outcomes of patients with recurrent spontaneous abortion

**DOI:** 10.12669/pjms.41.5.10466

**Published:** 2025-05

**Authors:** Yanan Yang, Chong Liu, Na Han, Guohui Wang, Wei Zhao, Jiayin Shen

**Affiliations:** 1 Yanan Yang Department of Nursing, Hebei Research Institute for Family Planning Science and Technology, Shijiazhuang 050000, Hebei, China; 2 Chong Liu Department of Obstetrics and Gynecology, Hebei Research Institute for Family Planning Science and Technology, Shijiazhuang 050000, Hebei, China; 3 Na Han Hemodialysis Room, Langfang TCM Hospital, Langfang 065000, Hebei, China; 4 Guohui Wang Department of Anesthesiology, Yanshan People’s Hospital, Cangzhou 061000, Hebei, China; 5 Wei Zhao Department of Anesthesiology, Yanshan People’s Hospital, Cangzhou 061000, Hebei, China; 6 Jiayin Shen Department of Obstetrics and Gynecology, Langfang TCM Hospital, Langfang 065000, Hebei, China

**Keywords:** Individualized psychological Intervention, Recurrent spontaneous abortion, Psychological state, Pregnancy outcomes

## Abstract

**Objective:**

To investigate the effects of individualized psychological interventions on the psychological state and pregnancy outcomes of patients experiencing recurrent spontaneous abortion (RSA).

**Methods:**

This was a retrospective study. Eighty-eight patients with RSA treated at Langfang TCM Hospital from December 2022 to December 2023 were selected as study subjects. They were randomly divided into a control group, which received standard nursing care, and an intervention group, which underwent individualized psychological interventions. The psychological states and pregnancy outcomes of patients in both groups were compared through assessments of anxiety and depression scores, quality of life, recurrence rate of miscarriage, newborn Apgar scores, and patient satisfaction.

**Results:**

Before the intervention, no significant difference was observed in the baseline characteristics between the two groups(P>0.05). After the intervention, the Self-Rating Anxiety Scale (SAS) and Self-Rating Depression Scale (SDS) scores in the intervention group were significantly lower than those in the control group, respectively. The quality of life scores were all higher in the intervention group than in the control group. The recurrence rate of miscarriage in the intervention group (4.55%) was significantly lower than that in the control group (18.18%). The Apgar scores of newborns at birth, one minute after birth, and five minutes after birth were all higher in the intervention group. The satisfaction rate of the intervention group was significantly higher than that of the control group, with all differences being statistically significant(*P*<0.05).

**Conclusion:**

Individualized psychological interventions can effectively improve the psychological state and pregnancy outcomes of patients with recurrent spontaneous abortion.

## INTRODUCTION

Recurrent spontaneous abortion (RSA), commonly delineated as the occurrence of three or more consecutive miscarriages before the 20th week of gestation with the same partner^1^,^2^, represents a prevalent pregnancy complication. It affects 1%-5% of women of reproductive age^3^,^4^, with its incidence rate gradually increasing.^5^ The etiology of RSA remains multifaceted, with no identifiable cause in 50% of cases.^6^ Most patients with recurrent miscarriage endure considerable personal, familial, and societal stress, often leading to anxiety and depression.^7^ Anxiety and depression are recognized as significant risk factors for miscarriage.

The absence of effective psychological interventions exacerbates the physical and mental strain on the patient, potentially culminating in unfavorable pregnancy outcomes.^8^,^9^ Psychological interventions have gained widespread recognition and application in developed countries in Europe and America.^10^-^12^ The psychological states of different patients or the same patient at different stages vary significantly. This study endeavorsed to tailor psychological intervention strategies to the unique psychological profile of each patient, aimed to ameliorate their psychological state and achieve optimal pregnancy outcomes.

## METHODS

This was a retrospective study. Eighty-eight patients with recurrent spontaneous abortion (RSA) admitted to Langfang TCM Hospital from December 2022 to December 2023 were selected as study subjects by the principle of random sampling. Based on the nursing approach, patients were divided into a control group and an intervention group, with 44 individuals in each group.

### Ethical Approval:

The study was approved by the Institutional Ethics Committee of Langfang TCM Hospital (No.: 2023-123; date: December 1^st^, 2023), and written informed consent was obtained from all participants.

### Inclusion criteria:

- Patients diagnosed with RPL based on medical history and relevant examinations after admission.
- Those with ability to communicate normally.
- Those with miscarriage gestational weeks <20 weeks.
- Those who have volunteered themselves and their families to participate in the research project and have signed the relevant documents.
- Those with no other pregnancy diseases, cardiopulmonary diseases, or leukemia, etc.

### Exclusion criteria:

- Patients with mental illness or unable to communicate normally.
- Those with severe diseases such as malignant tumors.
- Those in the pre-pregnancy, pregnancy, or lactation period.
- Those who allergic to the drugs used in this study.

The ages ranged from 20 to 37 years, with three to five pregnancies on average. The average age in the control group was 28.43±5.66 years, with an average of 3.73±0.82 pregnancies; the intervention group consisted of 27 males and females each, with an average age of 28.80±5.33 years and an average of 3.75±0.81 pregnancies. No statistically significant difference was observed in the general data between the two groups of patients with recurrent miscarriages (*P*>0.05).

### Methods Control Group:

Standard nursing care interventions were applied.

The gestational status and uterine contractions of the patients were meticulously documented, followed by vigilant monitoring throughout the subsequent treatment process to ensure the timely implementation of appropriate therapeutic interventions.

In response to the patients’ various diagnostic indicators, interventions were made in their dietary habits, daily routines, and occupational activities to mitigate excessive fatigue, thereby reducing the risk of recurrent miscarriage.

Patients were educated on the fundamental knowledge of recurrent miscarriage, and their inquiries were addressed, enhancing their understanding and awareness of the condition.

### Intervention Group:

On top of standard nursing care interventions, an individualized psychological intervention plan was developed for patients.

Before the treatment started, the intervention group patients were scored for anxiety (SAS) and depression (SDS), and an individualized psychological intervention plan was formulated based on the scores;

Patients and their partners were educated on recurrent miscarriage to enhance their and their families’ awareness of recurrent spontaneous abortion (RSA), emphasizing the adverse impact of psychological stress on pregnancy outcomes, strengthening the partner’s role in psychological counseling in daily life, creating a relaxed family atmosphere, and reducing the patient’s stress from self and family environment;

One-on-one psychological counseling was provided for patients based on their psychological state, once to twice a week; (4) A WeChat group was established to answer common questions daily, enhance patient communication, and eliminate negative emotions. The two groups of patients were followed up for six months.

### Observation indicators:

### Comparison of anxiety and depression scores.

The Self-Rating Anxiety Scale (SAS) and Self-Rating Depression Scale (SDS) were employed to score the anxiety and depression levels of both groups.^13^ SAS scores below 50 and SDS scores below 53 are considered normal, indicating no anxiety. Scores were rated before and after treatment for both groups, with higher scores indicating more severe anxiety or depression.

### Comparison of quality of life:

A quality-of-life questionnaire for patients with recurrent miscarriage was devised by the medical team, covering seven dimensions: physical functioning, role physical, social functioning, emotional role, mental health, vitality, and bodily pain, with each dimension scored from 0 to 100. Higher scores indicate better quality of life.

### Comparison of the recurrence rate of miscarriage:

The number of patients experiencing recurrent miscarriages in both groups was statistically analyzed.

### Comparison of newborn Apgar scores:

The Apgar scores of newborns at birth, one minute after birth, and five minutes after birth were compared between the two groups, scoring newborns’ skin color, heart rate, respiration, muscle tone, and response to stimuli on a scale of 0, 1, or 2, for a total of 10 points. Higher scores indicate better newborn conditions.^14^

### Patient satisfaction:

A satisfaction questionnaire was customized by the medical team based on relevant literature from CNKI, covering treatment plan satisfaction, nursing communication skills, responsibility, timeliness, and effectiveness of problem-solving, among other aspects. The total score is out of 100, with scores below 60 indicating dissatisfaction, 60 to 79 indicating general satisfaction, and 80 or above indicating satisfaction. All the questionnaire was simply handed over to the participants to fill themselves, for patients under 18 years of age, satisfaction was jointly completed by the patient and their family.

### Statistical analysis:

All data were statistically analyzed using the SPSS 22.0 software(SPSS Inc., Chicago, IL, USA). Measurement data were expressed as (*χ̅±S*), and *t* test was used for comparison between groups. Enumeration data were expressed as percentage (%), and the chi-squared test (χ^2^) was used for data analysis. A *P*-value <0.05 was considered a statistically significant difference.

## RESULTS

No statistically significant difference in the Self-Rating Anxiety Scale (SAS) and Self-Rating Depression Scale (SDS) scores between the two groups before intervention (*P*>0.05). Table-I. Utilizing different psychological intervention methods, both groups showed a decrease in SAS and SDS scores, with the intervention group exhibiting significantly lower scores than the control group, with a statistically significant difference (*P*<0.01). This suggests that individualized psychological intervention can effectively improve the psychological state of patients with recurrent spontaneous abortion.

**Table-I:** Comparison of SAS and SDS scores (N=44,`χ±s, points).

Group	SAS score		SDS	
	Pre-intervention	Post-intervention	Pre-intervention	Post-intervention
Control group	57.80±3.67	46.91±3.67	56.57±4.44	48.20±4.73
Intervention group	57.39±3.29	39.27±6.31	57.59±4.22	31.43±6.89
*t*	0.550	6.938	-1.108	13.311
*P*	0.584	1.7137E-9	0.271	1.4484E-21

After psychological intervention, the intervention group’s scores in physical functioning, role physical, social functioning, emotional role, mental health, vitality, and bodily pain were all significantly higher than those of the control group (*P*<0.01) indicating that individualized psychological intervention can effectively enhance the quality of life for patients with recurrent spontaneous abortion. Table-II.

**Table-II:** Comparison of quality of life (N=44, *χ̅±S*, points).

Quality of life	Control group		Intervention group	
	Before treatment	After treatment	Before treatment	After treatment
Physical functioning	74.36±4.21	76.86±2.48	74.32±4.12	79.34±3.09^a^
Role physical	58.84±3.76	63.82±3.87	58.77±3.87	70.02±3.06^a^
Social functioning	54.48±3.02	59.86±3.23	53.64±3.50	72.25±5.02^a^
Emotional role	46.64±5.49	52.20±6.64	46.45±5.72	64.57±4.75^a^
Mental health	43.86±6.46	49.70±4.05	43.91±6.82	60.70±5.47^a^
Vitality	43.82±6.47	52.66±3.17	43.32±6.71	61.39±4.33^a^
Bodily pain	61.73±4.57	63.77±3.05	60.95±3.99	72.80±3.45^a^

*Note:* Post-intervention comparison with the control group,

a P<0.01.

The recurrence rate of miscarriage within six months in the intervention group was significantly lower than that in the control group, with a statistically significant difference (*P*<0.05).Table-III.

**Table-III:** Comparison of recurrence rate of miscarriage (N=44, cases).

Miscarriage status	Control Group	Combined Group
Miscarriage	8	2
No miscarriage	36	42
Recurrence rate of miscarriage/%	18.18	4.55

*Note:* Comparison of acute episode rates between the control group and the combined group, *χ^2^*=4.062, P=0.044.

The Apgar scores of newborns at birth, one minute after birth, and five minutes after birth in the intervention group were significantly higher than those in the control group, with a statistically significant difference (*P*<0.01). Table-IV The satisfaction rate in the intervention group (90.91%) was significantly higher than that in the control group (70.45%), with a statistically significant difference (*P*<0.05). Table-V.

**Table-IV:** Comparison of newborn Apgar scores (N=44, *χ̅±S*, points).

Group	At birth	one minute after birth	five minutes after birth
Control group	5.57±1.66	7.11±1.35	8.91±0.86
Intervention group	7.05±2.17	9.16±1.08	9.84±0.37
*t*	-3.588	-7.854	-6.617
*P*	0.000570	1.0417E-11	1.2457E-8

**Table-V:** Comparison of patient satisfaction (N=44, cases/%).

Complications	Dissatisfied	General	Satisfied	Satisfaction (%)
Control group	4/9.09	8/18.18	32/72.73	90.91
Intervention group	13/29.55	11/25.00	20/45.45	70.45

*Note:* Comparison of patient satisfaction between the control group and the combined group, *χ^2^*=5.906, P=0.015.

## DISCUSSION

Before the intervention, baseline comparisons revealed no significant disparities in the indicators assessed between the two groups (*P*>0.05). Subsequent to the tailored interventions, the intervention group demonstrated a marked improvement in mental health, as evidenced by lower Self-Rating Anxiety Scale (SAS) scores (39.27±6.31) and Self-Rating Depression Scale (SDS) scores (31.43±6.89) compared to the control group (46.91±3.67, 48.20±4.73). This suggests a better psychological state. Additionally, the quality of life scores in the intervention group, including physical functioning, role physical, social functioning, emotional role, mental health, vitality, and bodily pain, were all higher than those in the control group.

The recurrence rate of miscarriage in the intervention group (4.55%) was significantly lower than that in the control group (18.18%). The Apgar scores of newborns at birth, one minute after birth, and five minutes after birth were all higher in the intervention group, indicating better pregnancy outcomes compared to the control group. Patient satisfaction within the intervention group (90.91%) also significantly exceeded that of the control group (70.45%), with all observed differences achieving statistical significance (*P*<0.05). Pregnancy loss, a significant concern in clinical practice, affects 15-25% of all pregnancies, with the majority of sporadic losses being attributed to random numerical chromosomal errors, particularly trisomies.^15^ Less than 5% of women will experience two consecutive miscarriages, and only 1% will suffer three or more, representing one of the unresolved challenges in reproductive medicine.^16^ Women with recurrent spontaneous abortion are prone to feelings of depression, anxiety, guilt, and anger, with the incidence of depression being approximately five times higher among these women, further impacting their pregnancy outcomes.^17^,^18^ Hence, effective psychological intervention is necessary for treatment.

To date, much of the literature has focused on the experiences of women, neglecting the significance of the male partner’s experience in miscarriage and the interaction between partners. This field is gradually shifting towards a couple-based approach, emphasizing the value of psychological counseling for the couple as a whole.^19^-^21^ Building upon standard care, this study incorporated knowledge dissemination and psychological interventions for partners, enhancing the involvement of patients’ partners in the treatment process. Individualized psychological intervention treatment plans were developed based on the specific circumstances of different patients and their partners, aiming to reduce anxiety and depression in partners and strengthen mutual support among couples. Addressing patients’ psychological health issues comprehensively in a clinical setting improves pregnancy outcomes.

### Limitations:

However, a small number of samples was the shortcoming of this study. In view of this, more samples should be included in future studies to further validate the findings of this study.

## CONCLUSIONS

Individualized psychological interventions can effectively improve the psychological state and pregnancy outcomes of patients with recurrent spontaneous abortion. The study highlights the profound psychological impact of recurrent spontaneous abortion on both men and women, pointing to the distinct experiences and the lack of adequate societal support and scholarly attention. This underscores an urgent need for expanded research into psychological health interventions, particularly for men affected by recurrent spontaneous abortion.

### Authors’ Contributions:

**YY** and **JS:** Carried out the studies, participated in collecting data, and drafted the manuscript, and are responsible and accountable for the accuracy or integrity of the work.

**CL, NH, GW** and **WZ:** Performed the statistical analysis and participated in its design. Critical Review.

All authors read and approved the final manuscript.

## References

[1] Toth B, Würfel W, Bohlmann M, Zschocke J, Rudnik-Schöneborn S, Nawroth F (2018). Recurrent Miscarriage:Diagnostic and Therapeutic Procedures. Guideline of the DGGG, OEGGG and SGGG (S2k-Level, AWMF Registry Number 015/050). Geburtshilfe Frauenheilkd.

[2] Sakar MN, Oglak SC (2020). Letrozole is superior to clomiphene citrate in ovulation induction in patients with polycystic ovary syndrome. Pak J Med Sci.

[3] Shi B, Chen J, Chen H, Lin W, Yang J, Chen Y (2022). Prediction of recurrent spontaneous abortion using evolutionary machine learning with joint self-adaptive sime mould algorithm. Comput Biol Med.

[4] La X, Wang W, Zhang M, Liang L (2021). Definition and Multiple Factors of Recurrent Spontaneous Abortion. Adv Exp Med Biol.

[5] Rasmark Roepke E, Matthiesen L, Rylance R, Christiansen OB (2017). Is the incidence of recurrent pregnancy loss increasing?A retrospective register-based study in Sweden. Acta Obstet Gynecol Scand.

[6] Vomstein K, Aulitzky A, Strobel L, Bohlmann M, Feil K, Rudnik-Schöneborn S (2021). Recurrent Spontaneous Miscarriage:a Comparison of International Guidelines. Geburtshilfe Frauenheilkd.

[7] Otani-Matsuura A, Sugiura-Ogasawara M, Ebara T, Matsuki T, Tamada H, Yamada Y (2022). Depression symptoms during pregnancy and postpartum in patients with recurrent pregnancy loss and infertility:The Japan environment and children's study. J Reprod Immunol.

[8] Quenby S, Gallos ID, Dhillon-Smith RK, Podesek M, Stephenson MD, Fisher J (2021). Miscarriage matters:the epidemiological, physical, psychological, and economic costs of early pregnancy loss. Lancet.

[9] Torabi M, Kazemi A, Abdishahshahani M (2019). Psychometric properties of revised version of the fertility adjustment scale in infertile couples undergoing assisted reproductive technology. Eur J Obstet Gynecol Reprod Biol.

[10] Bighelli I, Rodolico A, García-Mieres H, Pitschel-Walz G, Hansen WP, Schneider-Thoma J (2021). Psychosocial and psychological interventions for relapse prevention in schizophrenia:a systematic review and network meta-analysis. Lancet Psychiatry.

[11] Adib-Rad H, Basirat Z, Faramarzi M, Mostafazadeh A, Bijani A (2019). Psychological distress in women with recurrent spontaneous abortion:A case-control study. Turk J Obstet Gynecol.

[12] Hennessy M, Dennehy R, Meaney S, Linehan L, Devane D, Rice R (2021). Clinical practice guidelines for recurrent miscarriage in high-income countries:a systematic review. Reprod Biomed Online.

[13] Yue T, Li Q, Wang R, Liu Z, Guo M, Bai F (2020). Comparison of Hospital Anxiety and Depression Scale (HADS) and Zung Self-Rating Anxiety/Depression Scale (SAS/SDS) in Evaluating Anxiety and Depression in Patients with Psoriatic Arthritis. Dermatology.

[14] Abukari AS, Awuni N, Yakubu I, Mohammed S, Yakubu A, Yakubu S (2021). Factors associated with low fifth minute Apgar score in term and preterm singleton live births in a Ghanaian hospital. J Neonatal Nurs.

[15] Genovese HG, McQueen DB (2023). The prevalence of sporadic and recurrent pregnancy loss. Fertil Steril.

[16] Cao C, Bai S, Zhang J, Sun X, Meng A, Chen H (2022). Understanding recurrent pregnancy loss:recent advances on its etiology, clinical diagnosis, and management. Med Rev (2021).

[17] Voss P, Schick M, Langer L, Ainsworth A, Ditzen B, Strowitzki T (2020). Recurrent pregnancy loss:a shared stressor---couple-orientated psychological research findings. Fertil Steril.

[18] Jensen KHK, Krog MC, Koert E, Hedegaard S, Chonovitsch M, Schmidt L (2021). Meditation and mindfulness reduce perceived stress in women with recurrent pregnancy loss:a randomized controlled trial. Reprod Biomed Online.

[19] Ruderman RS, Yilmaz BD, McQueen DB (2020). Treating the couple:how recurrent pregnancy loss impacts the mental health of both partners. Fertil Steril.

[20] Horesh D, Nukrian M, Bialik Y (2018). To lose an unborn child:Post-traumatic stress disorder and major depressive disorder following pregnancy loss among Israeli women. Gen Hosp Psychiatry.

[21] Hedegaard S, Landersoe SK, Olsen LR, Krog MC, Kolte AM, Nielsen HS (2021). Stress and depression among women and men who have experienced recurrent pregnancy loss:focusing on both sexes. Reprod Biomed Online.

